# A feasible approach to smart remote health monitoring: Subscription-based model

**DOI:** 10.3389/fpubh.2023.1150455

**Published:** 2023-04-11

**Authors:** Sylvester Joanne Kirubakaran, Ashok Gunasekaran, D. Raveena Judie Dolly, D. J. Jagannath, J. Dinesh Peter

**Affiliations:** ^1^Department of Electronics and Communications Engineering, Karunya Institute of Technology and Sciences, Coimbatore, India; ^2^Department of Computer Science and Engineering, Karunya Institute of Technology and Sciences, Coimbatore, India

**Keywords:** telehealth, telemedicine, IoT, remote health monitoring, subscription

## 1. Introduction

Health care can benefit greatly from the Internet of Things devices, including improved outcomes and cost savings. Three patients with Parkinson's disease were able to save ~1,500 h of travel time, 100,000 km, and $37,000 by utilizing Telehealth in 2006, as part of an observational study (1). Based on the evidence that Telehealth is reliable for Parkinson's disease evaluations, it is a wonder if it would also be effective for patients in other medical fields (2). Following the deployment of Telehealth because of the recent COVID-19 pandemic, Telehealth is increasingly available in outpatient centers and mobile clinics.

The study by Deeb et al. (3) confirms that patient satisfaction with Telehealth visits was not significantly different from in-person appointments for outpatient movement disorders. Most respondents, especially for Telehealth visits, were satisfied with how they understood the care plan, were satisfied with the nurse's responsiveness and felt that the visit was sufficient. Generally, Telehealth patients were satisfied with the ease of using the technology during their visits (4). In light of this, Telehealth may not be as negative as commonly imagined, particularly given the challenges of technology of navigation.

Telemedicine is introduced for ambulatory care triage and treatment, which allows the protection of patients and doctors to avoid unnecessary exposure. In accordance with the Health Insurance Portability and Accountability Act (HIPAA), doctors can take advantage of online virtual meeting applications like Google meet, Microsoft Teams, and Skype for their consultations (5). Telemedicine health services are provided to all hospitals collectively to obtain and manage data to check the health status of each patient (6). In addition to improving patient outcomes, IoT can reduce costs by facilitating continuous monitoring and early intervention in chronic care, as well as supporting medication adherence (7).

## 2. Current trends

Using telemedicine has been beneficial beyond triage, allowing large healthcare providers to act quickly when local hospitals and health centers cannot meet demand (8). It was through telemedicine that both people, infected, and uninfected, received health information during this infectious pandemic. Telemedicine is being considered for chronic disease treatment, such as diabetes and congestive heart failure, where studies have shown similar results (9). Telemedicine has become more prevalent, prompting discussions about how it should be integrated into healthcare provider accreditation. New third-party methods have emerged in telemedicine because of increased use. Traditionally, telehealth was used in villages and far, out-of-reach locations only, but it's now used to expand the geographical influence of healthcare and promotion of accessibility (10). Telehealth includes communication between patients and providers *via* telephone, email, video chat, or conference (11). The proposed smart medicine box by Al-Mahmud et al. (12) comes with a wireless internet connection, and patients‘ are helped to receive care and interaction with the doctor without a face-to-face session. The data measured on the server is used for the doctor's observation. There are a number of Internet protocols that are commonly used on the Internet of Things for applications regarding healthcare (13). Some of the most common protocols discussed by the author include MQTT, which is a lightweight messaging protocol that is commonly used in IoT applications due to its low overhead and high performance.

The table summarizes key features of commercial smart health monitoring systems (14–18), including the types of vitals measured, data collection methods, data processing techniques, availability of mobile app interfaces, telehealth services, and artificial intelligence capabilities.

Javed et al. (19) present a “collaborative shared healthcare plan framework” that is very similar to the work done by Javed et al. (20) using smartphone sensors to track the activities of an individual and use that data to analyze daily routines through machine learning. The difference is that Javed et al. (19) suggests a framework based on collaboration between doctors, patients, and even their guardians for a shared plan for healthcare, an aspect through which it advocates for transparency. Javed et al. (19, 20) have use cases that are targeted and directed at specific individuals with a certain cognitive impairment like Dementia, ergo the solution doesn't appeal to the general mass. There are several benefits to cloud integration and big data in IoT healthcare systems (21). Cloud-based systems can store large volumes of data and make it available to authorized users from any location. This can be particularly useful in healthcare, where data may need to be accessed by multiple parties, such as patients, caregivers, and healthcare providers. Salloum and Tekli (22) have introduced a fuzzy logic-based nutrition and health monitoring system called “Personalized Intelligent Nutrition” using fuzzy reasoning to simulate the human thought process and intelligently assesses weight, calorie intake metrics, and the user's target goal body transformation progress and recommends steps to maintain or improve the said metrics. The paper by Tyagi et al. (23) explains the features of a sensor network using WBASN- a “wireless body area sensor network” that is placed on or near the body and is used to monitor various health metrics, such as vital signs or activity levels. These sensors can transmit data wirelessly to a central device, such as a smartphone or cloud platform, using technologies such as Bluetooth or Wi-Fi. The paper by Ibrahim and Zhuopeng (24) shows a patient health monitoring system, using the internet of things, by monitoring patients' heartbeat and body temperature. The recorded data is processed using Atmega 328 and data is transmitted using an external Wi-Fi module. The major demerit of that proposed solution is the usage of analog sensors for detecting body vitals such as LM35 and LM358 are prone to white noise and are not precise in recording vitals. Pardeshi et al. (25) demonstrates the use of a Raspberry Pi to monitor a patient's body temperature, blood pressure, egg, and heartbeat as part of an “internet of things” based health monitoring system. The author proposes to capture and send recorded data through Wi-Fi, Bluetooth, NFC, and LTE. The proposed solution by Adi and Kitagawa (26) uses MPS20N0040D-S type high precision MEMS pressure sensor to detect blood pressure. For amplification, the author proposed to use LM358N amplifier, and noise is regulated through a customized bandpass filter.

## 3. Impacts and challenges

The main challenges associated with telehealth include thorough physical patient examination limitations, potential data breaches, technical problems, and regulatory restrictions. Those who criticize telehealth point out that online communication can undervalue care continuity and are dangerous from a care perspective (27). To treat and diagnose, a virtual agent cannot conduct a complete and proper physical exam and history. The legal risks and consequences of telehealth should be familiar to healthcare providers.

Affordability comes into question, especially in lower-income countries, where medical healthcare facilities decide between life and death because of access to medicine and general quality of life. Diagnostics are readily available in 9.2% of primary facilities for healthcare, 19.1% of care settings, and 68.6% of hospitals in low-income countries. Products were available in 43 different countries in a range of 89.6%−1.9%. Results of the WHO Essential Diagnostic Lists and Priority Medical Device Initiatives can be used as benchmarks to measure progress toward implementing better guidelines (28).

In different countries and locations, obtaining licenses and maintaining them is difficult with telehealth due to the lack of multistate licensing. Physicians and physician assistants can practice telemedicine developed by the Federation of State Medical Boards (29). Providers remain subject to the licensing and authority discipline of medical boards of the state. Nevertheless, they share licensing and policy information and processes. In the event of a violation, the offender can be fined, jailed, or disqualified from receiving Medicare benefits (11). Regulations differ between states, requiring state-by-state analysis. De Witte et al. (30) have conducted a survey across Europe to understand the standing of several mental health professionals in the surge in online consultations because of the COVID-19 pandemic. Their results show that many of the consultants have had a positive experience and believed that the future of online telehealth consultations is not far and is inevitable, although their only reservation was about the security of the software and the privacy of patients. Cyberattacks such as Distributed Denial of Service (DDoS) attacks involve flooding a website or other online service with traffic from multiple sources and Medjacking, or the hijacking of a medical device, is also a type of cyberattack (11). Due to the use of online servers and databases by hospitals to maintain patient records, the risk of getting hacked and identities stolen have increased (31). Misinformation and denial of service due to cybersecurity breaches can be precarious to the lives of patients.

According to Table 1, remote medical check-up kits are already available, but they cannot be a substitute for preliminary medical tests performed by doctors at hospitals. For instance, none of the products described in the table prescribe an electronic stethoscope to measure heart sounds, which is a very vital test to be taken. The importance of the detection of heart sounds has been highlighted by Li et al. (32) because of the rise in cardiovascular diseases across the world. The stethoscope is a doctor's best friend that gives them a glimpse of their patient's health. The present solutions describe a product-based solution and are independent ventures. Not all patients can be expected to use the kit to test themselves and interpret the results. Even if doctors and general physicians prescribe these kits to their patients, they need to properly stay in touch with their patients and guide them remotely, which results in sporadic implementation. None of the proposed solutions discuss a systematic subscription-based model, with a medium of communication between the doctor and the patient that are service based and implemented through the hospital itself.

**Table 1:** Summary of the reviewed commercial smart health monitoring systems.

**S. no**	**Remote patient monitoring companies**	**Country**	**Method of collecting patient's vitals**	**Vitals collected**	**Method of collecting data and processing data**	**Mobile application interface**	**Telehealth services**	**AI**	**Mode of deployment**
1	GYANT	Portugal	None	None	Digitizes discrete patent's data in an integrated dashboard and analyses the uploaded data	Yes, for accessing patient's data and for telehealth services	Chat based and video based	Yes, only in vital survey and preliminary analysis	Standalone product/commercial (only software)
2	Chronisense Medical	Israel	Wearable smart watch	Blood pressure, heart rate, blood oxygen, ECG and blood glucose	Updates data in an integrated dashboard	Yes, for accessing patient's data	No	Yes, only in vital collection	Standalone product/commercial (only hardware)
3	100 Plus	United States	Individual IoT based devices	Blood pressure, heartrate, blood glucose and blood oxygen level	Updates data in an integrated dashboard	Yes, for accessing patient's data and for telehealth services	Chat based and video based	Yes	Standalone product/commercial (only software)
4	Vitls	United States	Disposable wearable sensor	Heart rate, respiration rate, blood oxygen level and body temperature	Updates data in an integrated dashboard	Yes, only for doctors and nurses	No	No	Standalone product/commercial (exclusively for hospitals)
5	ContinUse Biometrics (Cu-Bx™)	Israel	Contactless optical sensor	Heart rate and respiration rate	Updates data in an integrated car infotainment system	No	No	Yes, for capturing data	Standalone product/commercial (only software and exclusively for consumer automobiles)

## 4. Future directions

The recent rise in population has led to increased medical complications worldwide. The hospitals find themselves filled with patients waiting their turn before they see the doctor, especially with preliminary check-up tests. A solution can be devised that reduces the layover time between the patient and the doctor to provide an affordable solution to rudimentary medical check-ups.

A mobile app could form an interface for a medical kit that encompasses multiple basic medical check-up devices. The kit and the app could record the parameters, likely the heart sounds through an Electronic (E) Stethoscope, body temperature using a contactless infrared thermometer, a blood pressure machine, and an Electrocardiogram, and sends them to the doctor. This would help reduce the layover time between the doctor and the patient by avoiding the wastage of time and resources for rudimentary medical tests taken right before a doctor's appointment and allowing meeting through virtual mode. This helps improve the sanitary conditions of the public in general, especially when social distancing is needed in case the pandemic gets worse and provides mobile, user-friendly, and portable means to help with a diagnosis on the go. The kit could use a 4G hotspot so the user can connect to the kit through their mobile app even if there's no internet available. The medical kit could be issued by the hospitals to their regular patients through a subscription model instead of selling as a standalone kit so that the patient records could be easily accessed by the hospital and the virtual doctor meeting could be set up with ease. A mobile application can provide a user-friendly interface. It should include features like E-stethoscope recording, EKG, temperature measurement, blood pressure reading, BMI calculator, and pedometer. The cloud platform dashboard monitors several measured parameters that are available to the hospital and the users. The E-stethoscope records heart sounds with the app and offers the possibility to share them with the doctor *via* Gmail, WhatsApp or Telegram. The non-contact infrared thermometer can include a fever alarm for both the doctor and the user. The work by Salloum and Tekli (22) could inspire a nutrition tracker feature in the mobile app for the medical kit that could help the users with their fitness regime by analyzing their body mass index and the pedometer feature on the smartphone to recommend walking exercises.

**Figure 1:**
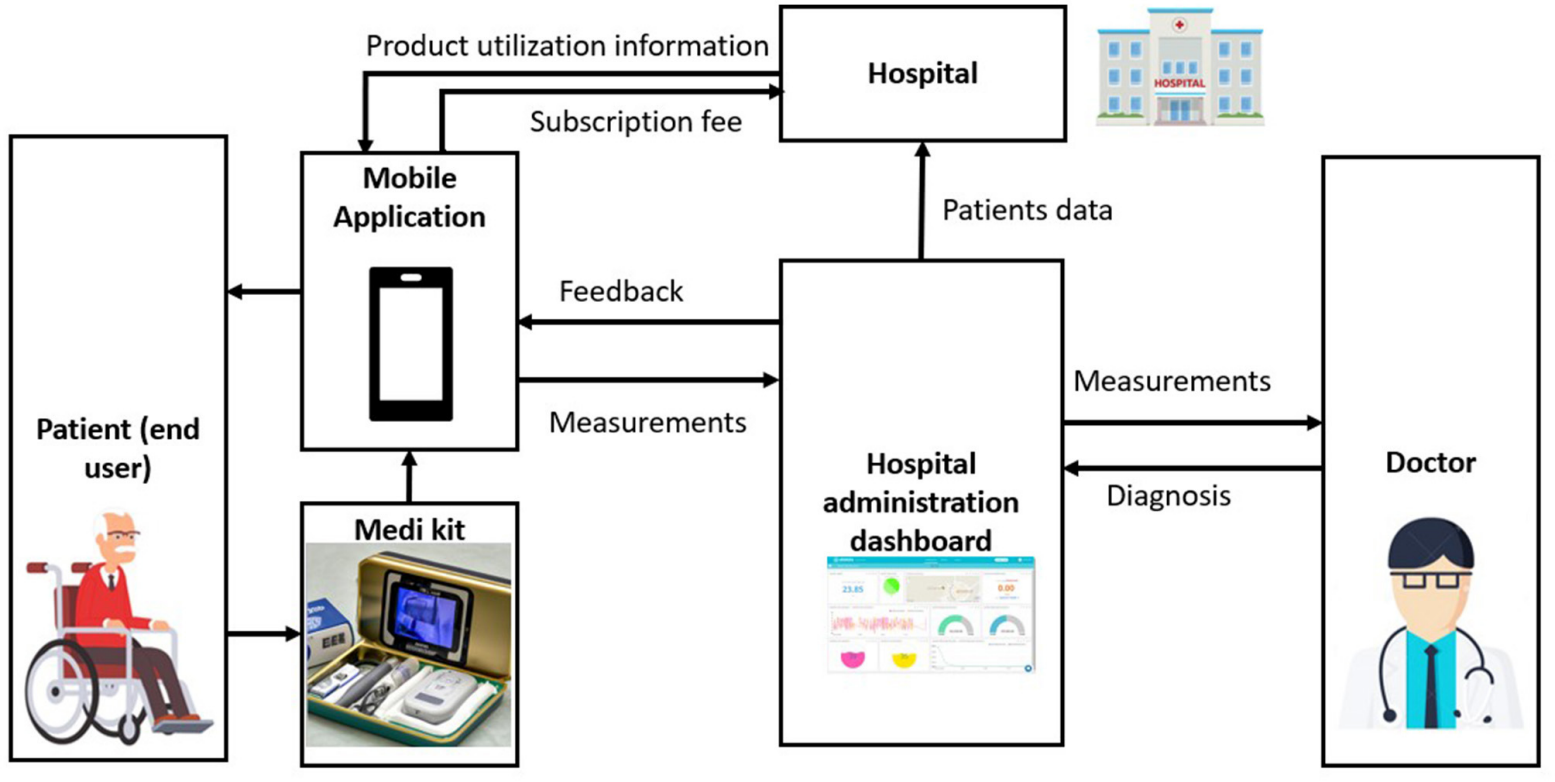
Flow diagram illustrates a service-based subscription model for Medi kit.

A subscription-based model of the system could ensure that a patient can easily contact a doctor. Figure 1 depicts a sample flow diagram that describes a service based subscription model. The hospital conglomerate may implement this by letting their patron patients subscribe to the service offered by the hospital. The kit could be purchased only from the hospital offering the service so that the buyer could use the kit to record their preliminary checkup tests on the mobile app and send them to the doctor at the subscribed hospital. The virtual consultation link is sent by the hospital administrator to the user/patient through the cloud platform switch. Huge hospital chains, insurance companies, and conglomerates can take up the initiative of smart remote health monitoring to ensure a systematic real-time patient data monitoring system is in effect. The doctors at these establishments could prescribe the proposed medical kit to their patients with a subscription plan to monitor their patients effectively and remotely with ease when it comes to general check-ups, follow-ups, or even intensive care at home. According to OneBill Software (33), the benefits of a subscription based model would greatly help with easy and quick access to various healthcare network providers, thus allowing for patients to obtain well suited care. OneBill Software (33) further vouches for a healthcare system that is proactive and engulfed with knowledge of consumers. Thus, the future is inevitably bound to incline toward the direction of remote health monitoring, which would be greatly benefitted by a subscription based health monitoring system. The presence of artificial intelligence (AI) capabilities in commercial smart health monitoring systems can further enhance the accuracy and reliability of the data analysis and recommendations provided to help doctors with faster diagnosis. AI can be used to detect patterns and trends in the data, predict potential health issues, and provide personalized health recommendations.

To protect against DDoS attacks and medjacking, it is important for healthcare organizations to implement robust cybersecurity measures, such as network firewalls and intrusion detection systems, to prevent unauthorized access to IoT devices. It is also important to regularly update software and firmware on IoT devices to ensure that they are protected against known vulnerabilities. Kaddoura et al. (31) have conceptualized a novel algorithm that is five times as fast as the ones in the market and memory efficient to identify malicious activities in the database and isolate them, simulating the precautions taken to avoid the spreading of COVID-19. Such an algorithm could bolster the safety of the database and revive it after a cyber-attack.

## Author contributions

SK and AG provided the main conceptual ideas, the proof outline, and worked out almost all of the technical details. DD, DJ, and JP worked on the manuscript. All authors contributed to the article and approved the submitted version.
